# Comparison of a standard environmental surface sampling method and a composite approach for select healthcare pathogens

**DOI:** 10.1017/ash.2023.325

**Published:** 2023-09-29

**Authors:** Monica Chan, Judith Noble-Wang, Laura Rose

## Abstract

**Background:** Hospital surfaces are known to contribute to the spread of healthcare-associated infections (HAIs). Environmental sampling is often performed to locate a reservoir or to evaluate intervention strategies in healthcare facilities. Composite sampling is commonly practiced in other fields of environmental sampling and involves collection of multiple samples combined entirely or partially to form a new sample. We compared a standard CDC surface whole-tool sampling method with a composite sampling approach. **Methods:** *Acinetobacter baumannii* (AB), *Klebsiella pneumoniae* that produce *K. pneumoniae* carbapenemase (KPC), vancomycin-resistant *Enterococcus faecalis* (VRE), methicillin-resistant *Staphylococcus aureus* (MRSA), and *Clostridioides difficile* spores were suspended in an artificial soil and deposited as 40 µL droplets (~104 CFU total) onto steel coupons of surface areas 323 cm^2^, 645 cm^2^, or 1,290 cm^2^ and dried for 2 hours. The surfaces were sampled with a single pass of a cellulose sponge— either the larger side of the sponge (face) or the smaller side of the sponge (edge)—and the optimal surface area was determined. Recovery from the optimal surface area with a single pass sampling was compared to the recovery using a standard CDC method in which all sides were used (ie, whole-tool method) to sample a standard area (645 cm^2^). Recovery was determined by culture and total CFU were determined for each optimal surface area. Theoretical composites were constructed using the mean total CFU of optimal surface area; 2×((face) + (edge)). Significance was set at *P* ≤ .05. **Results:** Total CFU recovery using the whole-tool method was significantly greater than the single pass sample recovery for MRSA (18,300 vs 16,600 CFU) and VRE (27,600 vs 26,400 CFU) (*P* < .05). When comparing the theoretical composite method to the standard whole-tool area (625 cm^2^), the theoretical composite total CFU was significantly greater than the whole-tool method for all organisms. For example, VRE recovery with the standard CDC whole-tool method was 27,600 CFU from 625 cm^2^, yet a theoretical composite approach recovered 79,800 CFU from an area of 1,290 cm^2^. **Conclusions:** Many factors influence recovery when sampling the environment, and composite sampling is a promising approach when sampling large surface areas. Using a theoretical composite of single-pass samples, the potential for improved detection with composite sampling was demonstrated. A composite sampling approach will reduce time and resources for sampling and sample processing, allowing larger surface areas to be investigated which will improve infection control strategies.

**Disclosures:** None

